# Exosomal miR-25-3p from mesenchymal stem cells alleviates myocardial infarction by targeting pro-apoptotic proteins and EZH2

**DOI:** 10.1038/s41419-020-2545-6

**Published:** 2020-05-05

**Authors:** Yi Peng, Ji-Ling Zhao, Zhi-Yong Peng, Wei-Fang Xu, Guo-Long Yu

**Affiliations:** Department of Cardiology, Xiangya Hospital, Central South University, Changsha, 410008 Hunan Province P.R. China

**Keywords:** Cell death, Cell death, Cardiovascular diseases, Cardiovascular diseases

## Abstract

Mesenchymal stem cell (MSC) therapy is a promising approach against myocardial infarction (MI). Studies have demonstrated that MSCs can communicate with other cells by secreting exosomes. In the present study, we aimed to identify exosomal microRNAs that might contribute to MSC-mediated cardioprotective effects. Primary cardiomyocytes were deprived of oxygen and glucose to mimic MI in vitro. For the animal model of MI, the left anterior descending artery was ligated for 1 h, followed by reperfusion for 12 h. MSC-derived exosomes were used to treat primary cardiomyocytes or mice. Cardioprotection-related microRNAs were determined, followed by target gene identification and functional studies with quantitative PCR, western blotting, MTT assay, flow cytometry assay, chromatin immunoprecipitation and dual-luciferase assay. We found that MSC co-culture reduced OGD-induced cardiomyocyte apoptosis and inflammatory responses. Cardioprotection was also observed upon treatment with MSC-derived exosomes in vitro and in vivo. In line with this, exosome uptake led to a significant increase in miR-25-3p in cardiomyocytes. Depletion of miR-25-3p in MSCs abolished the protective effects of exosomes. Mechanistically, miR-25-3p directly targeted the pro-apoptotic genes FASL and PTEN and reduced their protein levels. Moreover, miR-25-3p decreased the levels of EZH2 and H3K27me3, leading to derepression of the cardioprotective gene eNOS as well as the anti-inflammatory gene SOCS3. Inhibition of EZH2 or overexpression of miR-25-3p in cardiomyocytes was sufficient to confer cardioprotective effects in vitro and in vivo. We concluded that exosomal miR-25-3p from MSCs alleviated MI by targeting pro-apoptotic proteins and EZH2.

## Introduction

Myocardial infarction (MI) denotes the presence of myocardial injury due to myocardial ischaemia^1^. Despite the awareness of the high risk of death, the mortality rate of acute MI (AMI) is steadily increasing in China^2^. According to the Institute for Health Metrics and Evaluation, ischaemic heart disease is the second leading cause of death in China, with a 54% increase in the mortality rate from 2007 to 2017 (www.healthdata.org/china). Progress in therapeutic research is hence eagerly desirable. Mesenchymal stem cell (MSC) therapy for MI has been evaluated intensively in basic science and clinical trials. Pathologically, MI is characterized by myocardial cell death^1^ and pro-inflammatory responses^3,4^, which further exacerbate cardiac injury. MSCs, on the other hand, have been demonstrated to exert anti-apoptotic, regenerative and anti-inflammatory effects in animal models of MI^5–9^. Furthermore, a recent meta-analysis with data from 14 randomized placebo-controlled trials suggested that MSC therapy is safe and beneficial in terms of the improved left ventricular ejection fraction and regenerative outcomes^6^. These animal and clinical studies indicate that MSC treatment represents a promising approach for MI therapy. Therefore, revealing the mechanism by which MSCs exert protective effects in MI is of great significance.

MicroRNAs belong to a subclass of noncoding RNAs that post-transcriptionally regulate the expression of protein-coding genes. Dysregulated expression of microRNAs is frequently reported in cardiac tissues post MI^10,11^, and dysregulated microRNAs have been considered diagnostic biomarkers of MI^12,13^. In addition, the cardioprotective roles of microRNAs have drawn increasing interest^14–16^. For instance, microRNA-24 overexpression was demonstrated to counteract cardiomyocyte^17^ and endothelial cell^10^ apoptosis and initiate myocardial healing^18^ after MI. Likewise, microRNA-214 overexpression also appeared to be cardioprotective^19,20^. Nonetheless, delivery of microRNA to cardiac tissues is not an easy task, limiting the translation of microRNA from bench to clinic. In this regard, exosomes, which transfer cargos for cell–cell communication^21–23^, may be applied to efficiently deliver cargo exosomal microRNAs to target cells. Indeed, it was demonstrated that MSC-derived exosomes contain more than 50 microRNAs, such as microRNA-1246 and microRNA-25-3p^24^. Therefore, whether MSCs mediate cardioprotection via exosomes and other exosomal microRNAs is of great interest. Indeed, great attention has been drawn to the finding that microRNAs appear to be indispensable for the therapeutic effects of MSC-derived exosomes in different disease models^24,25^.

Enhancer of zest homologue 2 (EZH2) is a histone-lysine N-methyltransferase that is part of polycomb repressive complex 2 (PRC2). By the addition of methyl groups to histone H3 at lysine 27, EZH2 promotes the formation of heterochromatin and thereby represses gene expression^26^. In a mouse model of limb ischaemia, EZH2 was upregulated accompanied by a reduction in eNOS and BDNF expression^27^. Inhibition of EZH2 by 3-deazaneplanocin (DZNep) restored the levels of eNOS and BDNF, which then facilitated angiogenesis in ischaemic muscles. In addition, EZH2-mediated gene repression is involved in an exaggerated inflammatory response. It was reported that EZH2 deficiency promoted the expression of suppressor of cytokine signalling 3 (SOCS3) in macrophages and microglia^28^. SOCS3 in turn caused the degradation of Taf6, leading to suppression of nuclear factor κB (NF-κB) and its downstream pro-inflammatory responses. It should be noted that exaggerated inflammation is also evident after MI and deteriorates cardiac injury. EZH2 therefore might act as a critical modulator of MI by repressing eNOS and SOCS3.

In the current study, the roles of exosomes in MSC-mediated cardioprotective effects were explored with in vitro and in vivo in models of MI. Our study demonstrated that MSC-derived exosomal miR-25-3p targeted pro-apoptotic genes to facilitate cardiomyocyte survival and targeted EZH2, thereby disinhibiting the expression of SOCS3, leading to suppression of inflammation post MI.

## Materials and methods

### Culture of primary cardiomyocytes

Primary cardiomyocytes were cultured and adopted for in vitro studies. All animal studies were approved by the animal ethics committees of Xiangya Hospital, Central South University (approval number: 201503435). Cardiomyocytes were isolated from adult BALB/c mice (6–8 weeks old) according to a published protocol^29^. Heparin (5 IU/g body weight) was administered via intraperitoneal injection before heart isolation. The heart was then perfused with perfusion buffer (in mM; NaCl, 120; KCl, 5.4; Na_2_HPO_4_.7H_2_O, 0.33; MgSO_4_.7H_2_0, 246.48; taurine, 125.1; butanedione monoxide, 101.1; HEPES, 238.3; glucose, 180.16; EGTA, 0.4; insulin, 5 × 10^−5^ U/L) and digestion buffer (perfusion buffer without EGTA and with CaCl_2_, 0.3 mM; protease XIV, 0.2 mg/mL; collagenase II, 2.4 mg/mL). The ventricles were isolated, minced with forceps and dissociated by pipetting. Then, the mixtures were filtered to obtain a single-cell suspension, followed by two rounds of gravity sedimentation to remove fibroblasts and endothelial cells in the supernatant. The purified cardiomyocytes were seeded in laminin (10 μg/mL)-coated culture plates and maintained in Dulbecco’s modified Eagle’s medium (DMEM, Gibco, Grand Island, NY, US) supplemented with 10% heat-inactivated foetal bovine serum (FBS, Gibco, Grand Island, NY, US) and 1% penicillin-streptomycin solution (Gibco, Grand Island, NY, US) in a humidified atmosphere of 5% CO_2_ at 37 °C. Cells were tested without contamination with mycoplasma.

### Culture of primary MSCs

MSCs were isolated from BALB/c mice (6–8 weeks old) according to a published protocol^30^. Bone marrow was flushed out and collected from the tibia and femur with a 10 mL syringe. The cell suspension was filtered through a 70-mm filter mesh and seeded in culture dishes at a density of 25 × 10^6^ cells/mL with complete DMEM. After 3 h, the medium was replaced with fresh complete DMEM to remove haematopoietic cell lineages. Then, the medium was replaced with fresh complete DMEM every 8 h for up to 72 h of initial culture. When the remaining adherent cells reached 65–70% confluence (<2 weeks), trypsin solution (0.25%, with 1 mM EDTA) was added for 2 min at room temperature. The floating cells, i.e., MSCs, were collected for further culture. For MSC and cardiomyocyte co-culture experiments, cardiomyocytes were seeded on the bottom of each well, and MSCs were maintained in the cell culture inserts of the plate. Cells were tested without contamination with mycoplasma.

### Transfection

Short hairpin RNA (shRNA) targeting SOCS3 and its scramble control were designed by GenePharma (Shanghai, China). Oligos encoding the shRNA were then synthesized and inserted into the pGPH1 vector (GenePharma, Shanghai, China). MiR-25-3p mimics, inhibitors and their respective scramble control oligos were obtained from GenePharma (Shanghai, China). Cells were seeded onto 6-well plates. For transfection, the indicated plasmids and Lipofectamine 3000 (3 µL/well) were mixed and added to cells. For miRNA studies, 25 pmol of microRNA mimics/inhibitor or their scramble controls (NC) was mixed with 7.5 µL of Lipofectamine RNAiMAX reagent (Thermo Scientific, San Jose, CA, USA) and used for transfection. All the transfected cells were then cultured for 48 h before further assays unless indicated otherwise.

### Exosome isolation and characterization

Exosomes were enriched from MSC culture media with Total Exosome Isolation Reagent (#4478359, Thermo Scientific, San Jose, CA, USA). MSCs were cultured in DMEM containing 10% exosome-depleted FBS (#A2720803, Thermo Scientific, San Jose, CA, USA). The culture media were collected and centrifuged at 2000 × *g* for 30 min to remove cells and debris. The supernatant was transferred to a new tube and mixed with isolation reagent (v:v = 2:1) by vortexing thoroughly. The mixed solution was incubated at 4 °C overnight and then centrifuged at 10,000 × *g* for 1 at 4 °C. The pellet consisting of total exosomes was resuspended in PBS and ready for use. For morphology visualization, freshly isolated exosomes were resuspended in 2% paraformaldehyde in cold PBS. Then, exosomes were mounted on copper grids, fixed with 1% glutaraldehyde in PBS, negatively stained with uranyl-oxalate solution (pH 7) for 5 min, and embedded in methylcellulose solution. A transmission electron microscope was used to visualize exosomes. Size and size distribution profile of isolated exosomes was evaluated using a NanoSight NS500 instrument (NanoSight Technology, Malvern, UK). To further characterize the exosomes, western blotting was performed to detect the levels of exosome markers, i.e., HSP70, CD63 and CD9. Briefly, exosomes were lysed by RIPA buffer (NaCl, 150 mM; Triton X-100, 1%; sodium deoxycholate, 0.5%; SDS, 0.1%; Tris, 50 mM, pH 8.0) supplemented with protease and phosphatase inhibitor cocktails (#5872, Cell Signaling Technology, Danvers, MA, USA). The proteins were then resolved and visualized as described in the Western blotting section.

### Detection of exosomes uptake by cardiomyocytes

Isolated exosomes were incubated with 3.3 μL of Alexa FlourTM 488 C5 Maleimide (200 μg/mL, A10254, Thermo Scientific, San Jose, CA, USA) for 1 h at room temperature. The labelling was disturbed by passing through the exosome spin column (MW3000, 4484449, Thermo Scientific, San Jose, CA, USA), according to manufacturer’s instruction. The labelled exosomes were washed out and resuspended with 1 mL of serum free OptiMEM (31985088, Thermo Scientific, San Jose, CA, USA). For each well in a 4-well plate, 250 μL labelled exosomes were incubated with primary cardiomyocytes in the standard cell culture condition for 4 h at 37 °C. Cardiomyocytes were then counterstained with CellTracker Deep Red dye and mounted with ProLong Gold antifade mountants without DAPI (#P36934, Thermo Scientific, San Jose, CA, USA). The cells co-labelling with Maleimide (green) and cell tracker (red) under the confocal microscope were considered as positive cells containing exosomes.

### In vitro oxygen-glucose deprivation (OGD) model

Primary cardiomyocytes were cultured with glucose-free DMEM (#11966025, Thermo Scientific, San Jose, CA, USA) in an anaerobic chamber (1% O_2_, 5% CO_2_) at 37 °C for the indicated hours to induce ischaemic injury. For exosome treatment, cells were treated with exosomes 6 h after OGD treatment at concentrations of 50 μg/ml exosomes.

### MTT assay

Cardiomyocytes were seeded onto 96-well plates at a density of 5 × 10^3^ cells/well and treated as specified in the Results section. At the time point of the assay, 10 µL of MTT solution (Sigma-Aldrich, St. Louis, MO, USA) in PBS (5 mg/mL) was added to each well and incubated in the cell culture incubator for 3 h. The supernatant was removed carefully. The formazan crystals were then dissolved in 100 µL of dimethyl sulfoxide (DMSO, Sigma-Aldrich, St. Louis, MO, USA). Cell viability in each well was determined by optical density measurement at 490 nm.

### Annexin V/propidium iodide (PI) apoptosis assay

Cardiomyocytes were seeded onto 12-well plates at a density of 1 × 10^5^ cells/well. After treatment as specified in the Results section, the cells were trypsinized and harvested for staining using the Annexin V-FITC/PI Detection Kit, according to the manufacturer’s instructions (Sigma-Aldrich, St. Louis, MO, USA). Cells were analysed by flow cytometry (Becton-Dickinson, Franklin Lakes, NJ, US). The FITC + /PI− fraction and FITC + /PI + fraction were considered apoptotic cells (early and late apoptosis, respectively).

### Western blotting assay

Total protein was extracted with cell lysis buffer (50 mM Tris, 150 mM NaCl, 1% NP-40, 1 mM EDTA, pH 7.6) containing a cocktail of protease and phosphatase inhibitors. The protein concentration was determined using a Pierce BCA protein assay kit (San Jose, CA, USA) according to the manufacturer’s instructions. Samples (30 µg protein/lane) were separated by SDS-PAGE and then transferred onto PVDF membranes (0.22 µm pore, Roche). After blocking with TBST buffer (20 mM Tris, 137 mM NaCl, 0.1% Tween-20, pH 8.0) containing 5% non-fat milk, the membranes were incubated with primary antibodies against FASL (#AB16982), PTEN (#9188), EZH2 (#5246), H3K27me3 (#9733), histone H3 (#4499), eNOS (#32027), SOCS3 (#52113), p-p65 (#3033), p65 (#8242), p-IκB (#2859), COX-2 (#12282) and β-actin (#3700) overnight at 4 °C. Except for anti-FASL antibody, all the primary antibodies were ordered from Cell Signaling Technology (Danvers, MA, USA) and diluted 1:1000. Anti-FASL antibody was obtained from Millipore (Burlington, MA, USA). Then, the membranes were incubated with secondary antibody (1:3000, Cell Signaling Technology, Danvers, MA, USA) for 1 h at room temperature. The protein bands were visualized using Immobilon Western Chemiluminescent HRP substrate (Millipore, Burlington, MA, USA). The proteins were quantified using Quantity One software (Bio-Rad Laboratories, Inc., Hercules, CA, USA).

### RNA isolation and quantitative PCR (qPCR)

Total RNA from cells was extracted with TRIzol reagent (Thermo Fisher Scientific, San Jose, CA, US) according to the manufacturer’s instructions. Briefly, cells were resuspended in 1 mL TRIzol, followed by total RNA extraction with 200 µL chloroform and RNA precipitation with 500 µL isopropanol. Complementary DNA (cDNA) was synthesized from 1 μg of total RNA using the PrimeScript RT reagent Kit (Takara, Dalian, CN). For microRNA extraction from exosomes, the miRNeasy Serum/Plasma Kit (QIAGEN, Germantown, MD, US) was used. QIAzolLysis Reagent (1 mL) was mixed with 200 µL exosome to allow exosome lysis at room temperature for 5 min. This was followed by 200 µL chloroform treatment and microRNA enrichment with a RNeasy MinElute spin column. A TaqMan microRNA reverse transcription kit (Thermo Fisher Scientific, San Jose, CA, US) was then used to reverse transcribe miRNA to cDNA. cDNAs were diluted 20-fold with ddH_2_O and used for qPCR with a SYBR Premix EX Ta kit (Takara, Dalian, CN) in an ABI 7500HT real-time PCR system (Thermo Fisher Scientific, San Jose, CA, US). The gene expression levels in all samples were normalized to U6 snRNA (for miRNA) or β-actin (for mRNA) expression levels using the 2^−ΔΔCt^ method. The primer sequences in the current study are listed in Table 1.

**Table 1:** Primers list.

Gene	Forward primer	Reverse primer
IL-1β	GCCACCTTTTGACAGTGATGAG	AAGGTCCACGGGAAAGACAC
IL-6	TGCAAGAGACTTCCATCCAG	TCCACGATTTCCCAGAGAAC
TNF-α	CCGATGGGTTGTACCTTGTC	TGGAAGACTCCTCCCAGGTA
eNOS	AGCATACCCCCACTTCTGTG	GAAGATATCTCGGGCAGCAG
SOCS3	GCTCCAAAAGCGAGTACCAG	TGACGCTCAACGTGAAGAAG
eNOS(CHIP)	CCAGGAGTTCTTGTATGTATGG	GGTCCTTCTGTGATGTGGC
SOCS3(CHIP)	CGCTTCGGGACTAGGTAGGA	AGAAACCGGGAAAAGCTCCC

### Chromatin Immunoprecipitation (ChIP)-qPCR assay

A ChIP-qPCR assay was performed to assess the binding of EZH2 or H3K27me3 to the transcription regulatory regions of the eNOS and SOCS3 genes. ChIP assays were conducted with ChIP-IT express kits (Active Motif, Shanghai, CN) according to the manufacturer’s instructions. Cardiomyocytes were seeded onto 10 cm dishes at a density of 1 × 10^7^ cells/dish and treated as specified in the Results section. After treatment, cells were cross-linked with formaldehyde and lysed for chromatin isolation. The fixed chromatin was then sheared by enzymatic shearing cocktail (provided in the kit), incubated with anti-EZH2 or anti-H3K27me3 and captured by protein G-conjugated magnetic beads. This was followed by chromatin elution, cross-linking reversal and proteinase K digestion. The products were then ready for qPCR using primers listed in Table 1.

### Dual-luciferase reporter assay

Reporter assays were performed to investigate the regulation of the FASL, PTEN or EZH2 3′UTR by miR-25. Oligos containing the predicted miR-25 binding sites on the FASL, PTEN or EZH2 3′UTR and their corresponding mutant forms were cloned into the pmirGLO vector (Promega, Madison, WI, US). miR-25 mimics were applied for miR-25 overexpression. The pRL vector was used as the transfection control. During the transfection process, miR-25 mimics, pmirGLO vectors and pRL vector (25:25:1) were co-transfected into HEK293 cells with Lipofectamine 3000 at a ratio of 1:3 (DNA: Lipofectamine). As a control, miR-25 scramble oligos were also used for co-transfection in a separate experiments as described above. miR-25 mimics or scramble control were obtained from GenePharma (Shanghai, China). After transfection for 48 h, firefly luciferase activity was determined and adjusted by Renilla luminescence using the assay kit according to the manufacturer’s instructions (Promega, Madison, WI, US).

### Mouse model of ischaemia-reperfusion (I/R) injury

All surgical procedures were approved by the animal ethics committees of Xiangya Hospital, Central South University (approval number: 201503435). 50 BALB/c mice (male, 20–22 g, 6–8 weeks) were randomly grouped (five groups, *n* = 10) to establish I/R injury models. The surgeries followed the procedures described previously (Zhaobin Xu, 2018). The investigator was blinded to the group allocation during the experiment. Briefly, thoracotomy was performed to expose the left anterior descending artery(LAD), which was tightly ligated together with PE-10 tubing for 1 h (ischaemia), and then released to allow reperfusion for 12 h. Mice which did not recovered from I/R surgery were excluded. To explore the therapeutic effects of MSC-derived exosomes, isolated exosomes (5 μg in 100 μL PBS) or PBS were injected into the border zone of the infarcted heart at three sites 30 min after ligation.

### Statistical analysis

All experiments were performed at least three times. Data are presented as the mean ± standard deviation (SD) based on three independent experiments. All statistical analyses were carried out using GraphPad Prism 6 (GraphPad Software, Inc., San Diego, CA, USA). All data were in a normal distribution, and variance was similar between the groups that are being statistically compared. Statistical evaluation was performed using Student’s *t* test (two-tailed) between two groups or one-way analysis of variance (ANOVA) followed by Tukey’s post hoc test for multiple comparisons. *P* < 0.05 was considered statistically significant in all cases.

## Results

### MSC co-culture reduced OGD-induced apoptosis and cytokine expression in cardiomyocytes

**Fig. 1:**
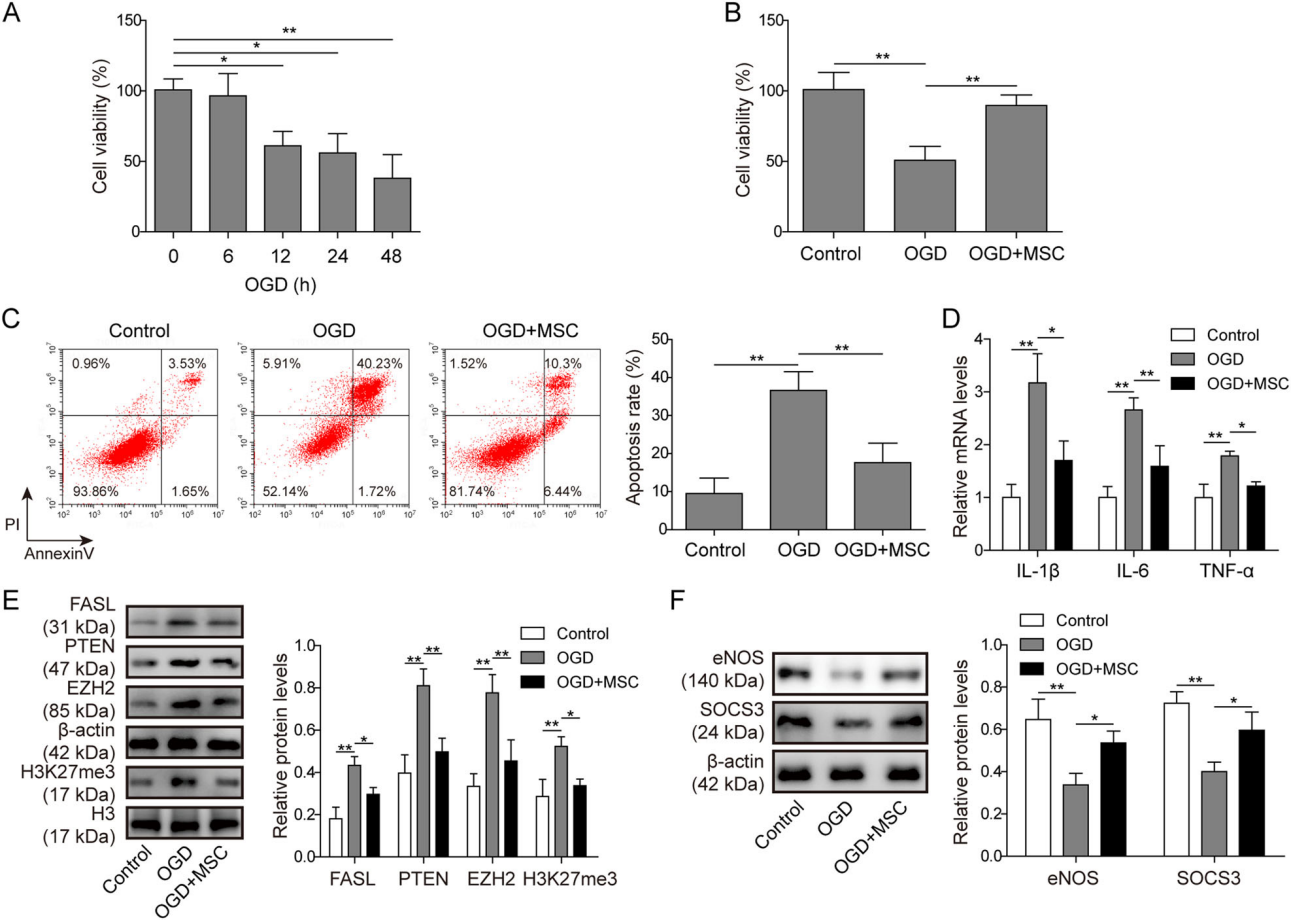
MSC co-culture reduced OGD-induced apoptosis and cytokine expression in cardiomyocytes. **a** MTT assay was conducted to detect cardiomyocyte viability at different time points after OGD. **b** Cell viability was detected in cardiomyocytes (control), cardiomyocytes deprived of oxygen and glucose (OGD) and co-cultures of cardiomyocytes and MSCs deprived of O/G (MSCs+OGD) by MTT assay. **c** Annexin V/PI staining was performed to detect cardiomyocyte apoptosis. **d** mRNA levels of IL-1β, IL-6 and TNF-α were assessed by qPCR. **e** The protein levels in the three groups were detected by western blotting. The statistical analysis is presented. **f** The levels of cardioprotective proteins were detected by western blotting. The statistical analysis is presented. Error bars represent the mean ± SD. *P < 0.05 and **P < 0.01.

The MTT assay demonstrated that cardiomyocyte viability gradually decreased in a time-dependent manner after OGD (Fig. 1a), indicative of a successful OGD insult. OGD for 12 h was chosen for subsequent in vitro experiments. To explore the protective actions of MSCs, cardiomyocytes were cultured alone or co-cultured with MSCs. As expected, OGD significantly reduced cardiomyocyte viability compared with control (Fig. 1b). Co-culture with MSCs, however, partially protected the cells from OGD-induced cell death. Similar findings were also obtained by the PI/Annexin V apoptotic assay. Compared with control, OGD caused dramatic accumulation of early and late apoptotic cardiomyocytes (Fig. 1c). The OGD-induced injury was significantly improved in the wells that were co-cultured with MSCs (Fig. 1c). Increase in pro-inflammatory cytokines is another characteristic of OGD-induced pathological alterations. qPCR demonstrated that OGD promoted the mRNA expression of IL-1β, IL-6 and TNF-α in cardiomyocytes compared with control cells (Fig. 1d). In the presence of MSCs, OGD-induced cytokine upregulation was greatly repressed (Fig. 1d). MSC-mediated cardioprotection was also evaluated by detecting OGD-related protein expression. As expected, OGD led to significantly higher levels of pro-apoptotic proteins, i.e., FASL and PTEN in cardiomyocytes compared with normal culture conditions (Fig. 1e). In addition, the protein levels of EZH2, a component of PRC2, and its target H3K27me3 were also elevated by OGD (Fig. 1e). Consistent with the cell death assay, MSC co-culture significantly suppressed OGD-induced upregulation of all these pro-apoptotic proteins (Fig. 1e). In contrast to the effect of OGD on cell death-related proteins, OGD decreased the protein levels of eNOS and SOCS3 (Fig. 1f), which have cardioprotective effects. MSC co-culture partially recovered the levels of these pro-survival proteins in cardiomyocytes after OGD (Fig. 1f).

### Uptake of MSC-derived exosomes led to microRNA increase in cardiomyocytes

**Fig. 2:**
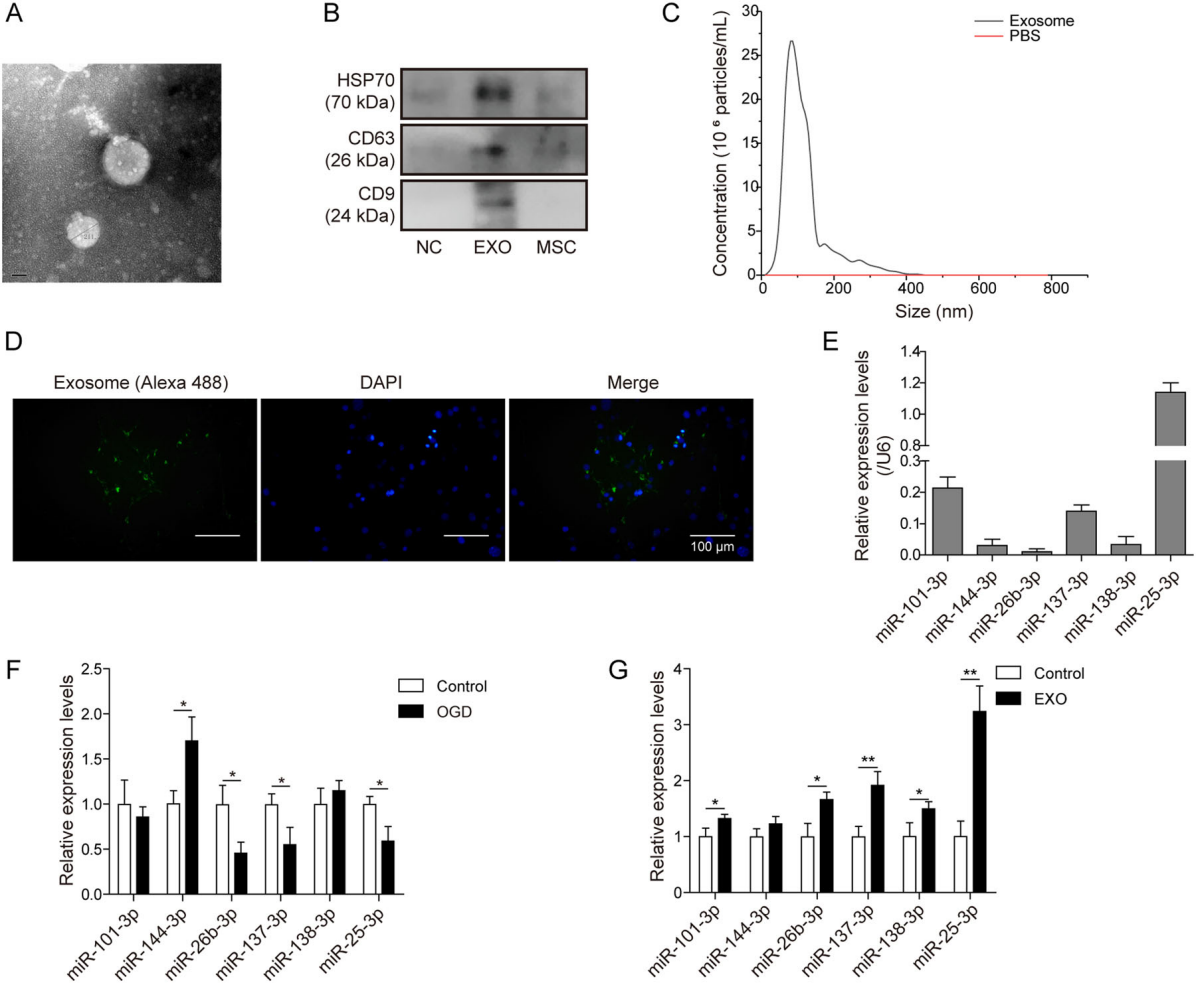
Uptake of MSC-derived exosomes led to microRNA increase in cardiomyocytes. **a** Representative images of exosomes from MSCs. **b** Exosomes were validated by assessing exosomal markers. **c** Size distribution of exosomes by nanosight. **d** Alexa 488-labelled exosomes (green) were observed in the cytoplasm of primary cardiomyocytes. **e** microRNA expression in isolated exosomes. **f** miR-25 in OGD-treated cardiomyocytes was detected by qPCR. **g** The levels of microRNAs in cardiomyocytes were detected by qPCR after exosome addition. Error bars represent the mean ± SD. *P < 0.05 and **P < 0.01. NC, negative control, denotes the exosome isolation reagent control; Exo, exosome.

Given that MSC-mediated cardioprotection is independent of direct cell–cell contact, we explored whether MSC-derived exosomes might play a role. Exosomes were isolated from the culture media of MSCs. TEM and western blotting confirmed that the purified product was highly enriched for exosomes (Fig. 2a, b). Size and size distribution showed a unimodal distribution of isolated particles with an average diameter ~100 nm, consistent with the definition of exosomes (Fig. 2c). Immunofluorescence staining showed that a strong Alexa 488 signal appeared in the cytoplasm of cardiomyocytes (Fig. 2d). As exosomes may act as sources of exogenous RNAs, the levels of miRNAs that were predicted to interact with EZH2 mRNAs (www.targetscan.org), were detected. qPCR showed that MSC-derived exosomes were enriched for a series of miRNAs, particularly miR-25-3p (Fig. 2e). The intracellular levels of miR-25-3p, miR-26b-3p and miR-137-3p were repressed in cardiomyocytes by OGD, while miR-144-3p was elevated, and miR-101-3p and miR-138-3p did not change (Fig. 2e). Exosomal miRNAs, in particular, miR-25-3p, were dramatically elevated after exosome incubation (Fig. 2g). This result implied that the addition of MSC-derived exosomes might provide increased miR-25-3p to modulate the molecular network of cardiomyocytes.

### MSC-derived exosomes conferred cardioprotection by supplementing miR-25-3p

**Fig. 3:**
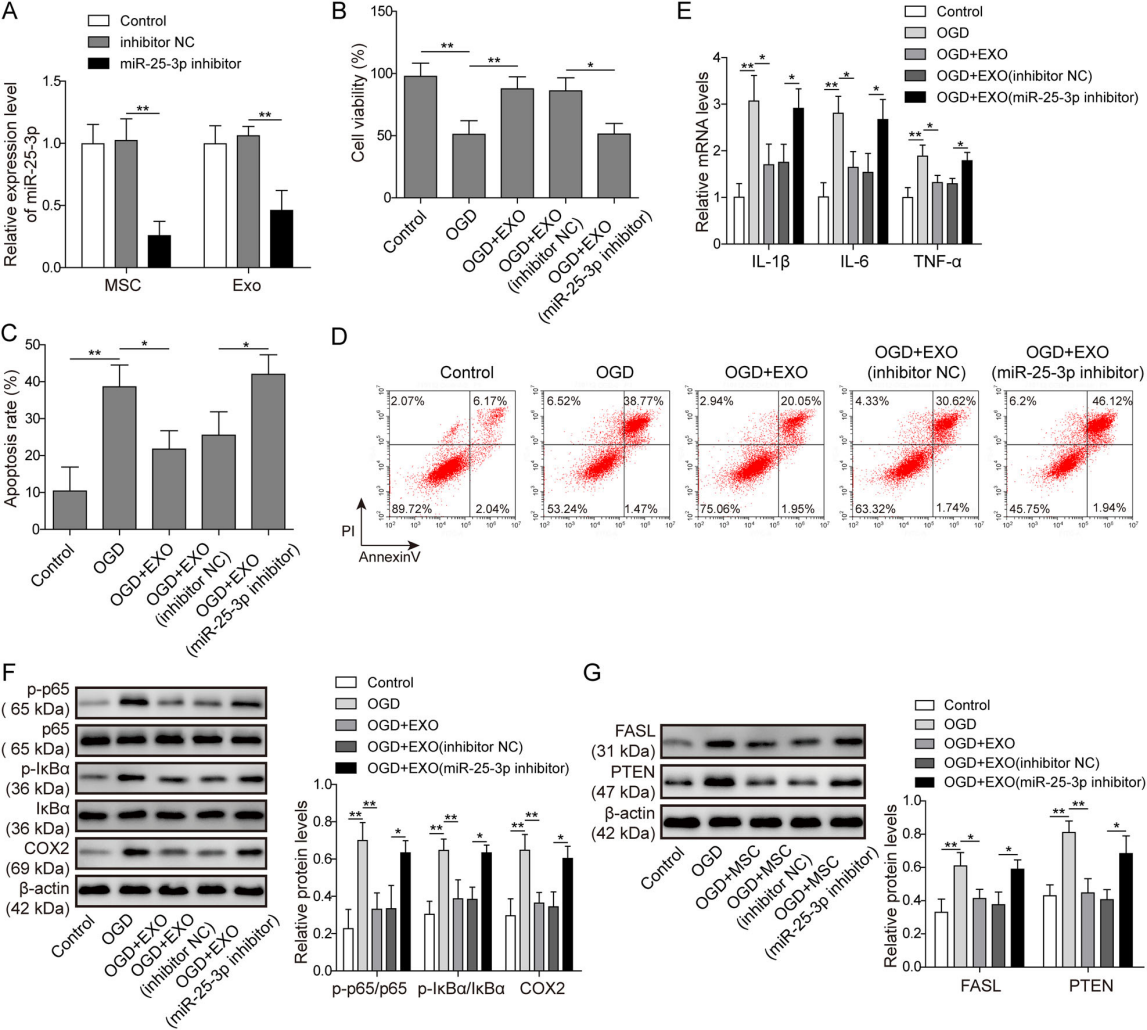
MSC-derived exosomes promoted cardioprotection by supplementing miR-25-3p. **a** The level of miR-25-3p was determined in exosomes from MSCs without treatment (control), treated with scramble (inhibitor NC) or treated with miR-25-3p specific inhibitor (miR-25-3p inhibitor). **b** Cell viability was detected by MTT assay. **c** Apoptosis was detected by flow cytometry after Annexin V/PI staining. **d** Statistical results of Fig. 3c. **e** The mRNA levels of IL-1β, IL-6 and TNF-α were assessed by qPCR. **f** Detection of proteins involved in pro-inflammatory pathways by western blotting. The statistical analysis is presented. **g** Detection of pro-apoptotic proteins. The statistical analysis is presented. Error bars represent the mean ± SD. *P < 0.05 and **P < 0.01.

To explore the roles of miR-25-3p in MSC-derived exosomes, a miRNA inhibitor was applied. As demonstrated in Fig. 3a, transfection with a miR-25-3p-specific inhibitor efficiently reduced the level of miR-25-3p in MSCs compared with the sham inhibitor or no treatment group. Notably, the exosomes isolated from MSCs transfected with miR-25-3p inhibitor also contained significantly lower levels of miR-25-3p than the exosomes from inhibitor NC-treated MSCs (Fig. 3a). Three groups of exosomes: those from MSCs without transfection (EXO), transfected with negative control inhibitor (EXO/inhibitor NC) or transfected with miR-25-3p inhibitor (EXO/miR-25-3p inhibitor) were isolated for a series of functional assays. Compared with the OGD alone group, MSC-derived EXO significantly increased cardiomyocyte viability after OGD in the MTT assay (Fig. 3b). A similar effect was also observed in the cells treated with EXO/inhibitor NC (Fig. 3b). However, cardioprotection was reversed when the cells were treated with an EXO/miR-25-3p inhibitor. The apoptosis rates were also assessed. As shown in Fig. 3c, d, while EXO or EXO/inhibitor NC significantly reduced OGD-induced early and late apoptosis, comparable apoptotic rates were observed in the cardiomyocytes treated with OGD alone or OGD + EXO/miR-25-3p. These differences between groups were also supported by the western blotting assay (Fig. 3g). OGD caused the accumulation of the pro-apoptotic proteins FASL and PTEN compared with the control treatment. Pre-treatment with EXO or EXO/inhibitor NC abolished this effect. However, depletion of miR-23-3p in exosomes failed to reduce the protein levels of FASL and PTEN compared with inhibitor NC group. These findings suggested that MSC-derived exosomes contributed to MSC-mediated anti-apoptotic effects and that miR-25-3p played a critical role in these effects. The effects of exosomes on cytokine expression were detected by qPCR. As expected, both EXO and EXO/inhibitor NC significantly reversed OGD-induced IL-1β, IL-6 and TNF-α upregulation (Fig. 3e). However, the exosome-mediated anti-inflammatory effects were abolished by pre-treatment with miR-25-3p inhibitor, as indicated by the comparison between the EXO/inhibitor NC and EXO/miR-25-3p inhibitor groups (Fig. 3e). The NF-κB pathway, which is highly associated with cytokine expression, was hence investigated using a western blotting assay. OGD promoted phosphorylation of p65 (NF-κB) and its upstream modulator IκB, while the total protein of p65 and IκB remained unchanged (Fig. 3f). Pre-treatment with EXO or EXO/inhibitor NC significantly reduced the phosphorylation of p65 and IκB after OGD. However, the inhibition of the NF-κB pathway in the EXO/miR-25-3p inhibitor group was diminished compared with that in the EXO/inhibitor NC group (Fig. 3f).

### Exosomes disinhibited EZH2-mediated repression of cardioprotective genes by introducing miR-25-3p

**Fig. 4:**
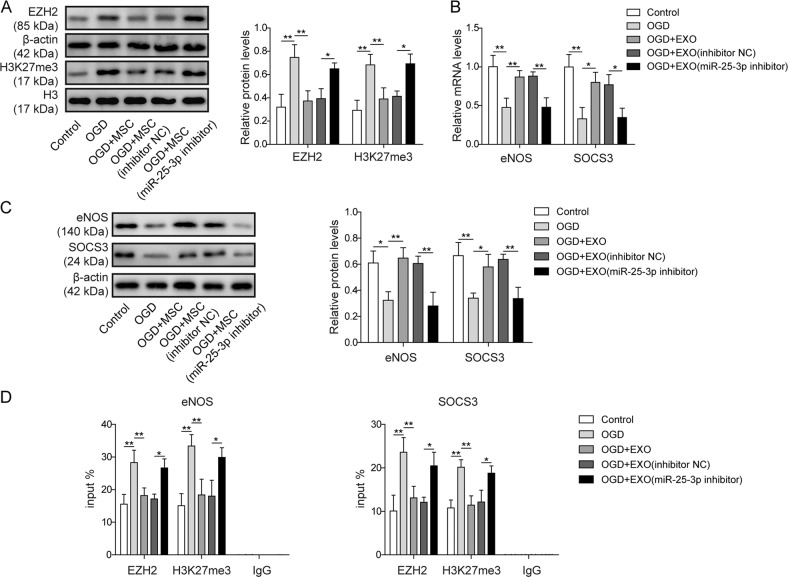
Exosomes disinhibited EZH2-mediated repression of cardioprotective genes by introducing miR-25-3p. **a** The levels of EZH2 and H3K27me3 were determined by western blotting. The statistical analysis is presented. **b** The mRNA levels of eNos, Bdnf and SOCS3 were determined by qPCR. **c** The protein levels of eNos and SOCS3 were determined by western blotting. The statistical analysis is presented. **d** ChIP was performed to detect the degree of EZH2 and H3K27me3 occupation in the regulatory regions of the eNOS and SOCS3 promoters. Error bars represent the mean ± SD. *P < 0.05 and **P < 0.01.

MSC co-culture may recover cardioprotective gene expression by inhibiting EZH2. We asked if miR-25-3p accounted for these effects. OGD increased the protein level of EZH2 and the trimethylation level at H3K27 (Fig. 4a). Consistent with the findings of MSC co-culture studies, EXO or EXO/inhibitor NC treatment was sufficient to suppress the OGD-induced increase in EZH2 and H3K27me3 (Fig. 4a). In contrast, depletion of miR-25-3p in the exosomes completely abolished this effect, as indicated by the comparison among OGD, OGD + EXO/inhibitor NC and OGD + EXO/miR-25-3p inhibitor (Fig. 4a). The alterations of EZH2 were further validated by detecting the transcription of the target genes eNOS and SOCS3. OGD significantly reduced the mRNA (Fig. 4b) and protein (Fig. 4c) levels of these cardioprotective genes. In contrast, EXO and EXO/inhibitor NC, but not EXO/miR-25-3p, recovered their expression (Fig. 4b, c). ChIP assays confirmed that both EZH2 and H3K27me3 were recruited to the promoter regions of eNOS and SOCS3 after OGD (Fig. 4d). Compared with that after OGD alone, recruitment of EZH2 and H3K27me3 to these promoter regions was significantly reduced after OGD + EXO or OGD + EXO/inhibitor NC treatment (Fig. 4d), while exosomes without miR-25-3p failed to block the binding of EZH2 and H3K27me3 to these promoter regions (Fig. 4d).

### miR-25-3p protected cardiomyocytes against OGD-induced apoptosis and increase in pro-inflammatory cytokines

**Fig. 5:**
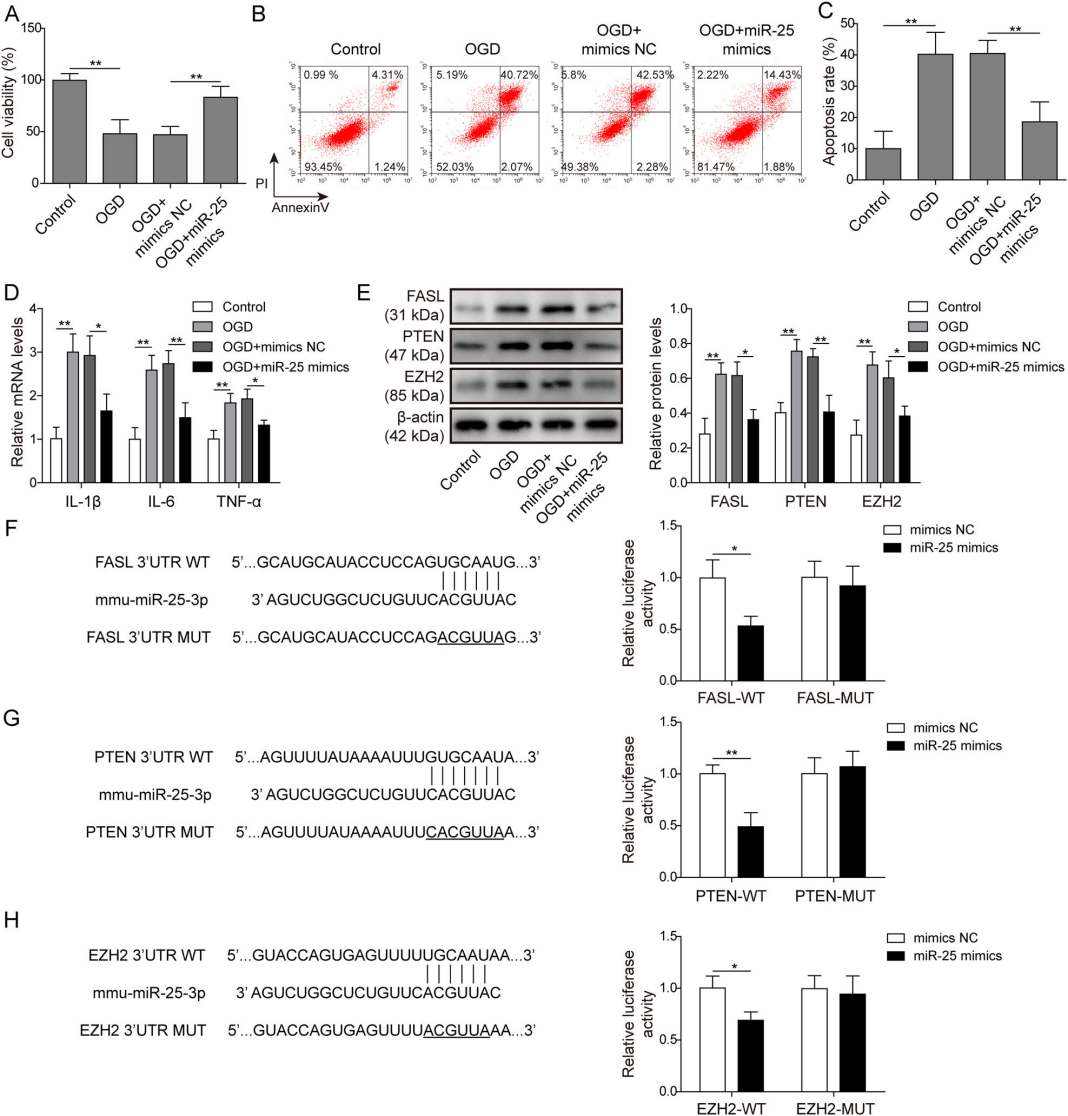
MiR-25-3p protected cardiomyocytes against OGD-induced apoptosis and upregulation of pro-inflammatory cytokines. **A** Cardiomyocytes without treatment were used as controls. OGD cells were deprived of oxygen and glucose for 12 h. OGD cells transfected with scramble oligos or miR-25 mimics are labelled as OGD + mimic NC and OGD + miR-25 mimic, respectively. The cell viability of each group was detected by MTT assay. **b** Apoptosis was detected by flow cytometry after annexin V/PI staining. **c** Statistical analysis of (**b**). (**D**) The mRNA levels of IL-1β, IL-6 and TNF-α were assessed by qPCR. **e** The protein levels of FASL, PTEN, H3K27me3 and EZH2 were determined by western blotting. The statistical analysis is presented. **f** Dual-luciferase assay in cells co-transfected with wild-type FASL 3′UTR and scramble microRNA (mimic NC) or miR-25 oligos (miR-25 mimic). Mutant FASL 3′UTR was also applied for co-transfection and dual-luciferase assays. **g** Dual-luciferase assay with PTEN 3′UTR. **h** Dual-luciferase assay with the EZH2 3′UTR. Error bars represent the mean ± SD. *P < 0.05 and **P < 0.01.

To further validate the cardioprotective effects of exosomal miR-25-3p, forced expression of miR-25-3p was applied to explore its molecular functions. In the MTT assay (Fig. 5a), transfection of the miR-25-3p mimic (miR-25 mimic) significantly impaired OGD-induced cytotoxicity compared with mimic NC transfection (Fig. 5a). In the PI/Annexin V assay, miR-25 mimic transfection significantly protected cardiomyocytes from OGD-induced apoptosis (OGD + mimic NC vs OGD + miR-25-3p mimic, Fig. 5b, c). Moreover, qPCR demonstrated that miR-25 mimic but not mimic NC blocked the upregulation of IL-1β, IL-6 and TNF-α after OGD (Fig. 5d), indicating that miR-25 exerted anti-inflammatory effects. The effects of miR-25-3p on apoptosis-related proteins were also assessed. Compared with mimic NC, miR-25-3p overexpression significantly attenuated OGD-induced upregulation of FASL, PTEN and EZH2 (Fig. 5e). In contrast, transfection of miR-25-3p inhibitor alone was sufficient to augment the mRNA levels of these pro-apoptotic modulators (Fig. S1). We then asked whether miR-25-3p might directly target the mRNA of these genes for degradation. Bioinformatic analysis revealed that the 3′UTRs of all these genes harboured putative binding sites of the miR-25-3p seed sequence (Fig. 5f–h). The 3′UTR fragments containing the putative binding sites (wild type, WT) or their mutant forms (Mut) were synthesized and cloned into the pmirGLO vector. Dual luciferase assays demonstrated that transfection of miR-25-3p mimic significantly reduced the luminescence of the three WT plasmids compared with mimic NC transfection (Fig. 5f–h). The repression effects of the miR-25-3p mimic were abolished when the Mut plasmids were co-transfected (Fig. 5f–h). Notably, the addition of exosomes containing miR-25-3p was sufficient to reduce the luminescence of the three WT plasmids compared with the vehicle control (Fig. S2). These results confirmed that FASL, PTEN and EZH2 were direct targets of miR-25-3p.

### Inhibition of EZH2 alleviated OGD-induced apoptosis and inflammation in cardiomyocytes at least partly through SOCS3

**Fig. 6:**
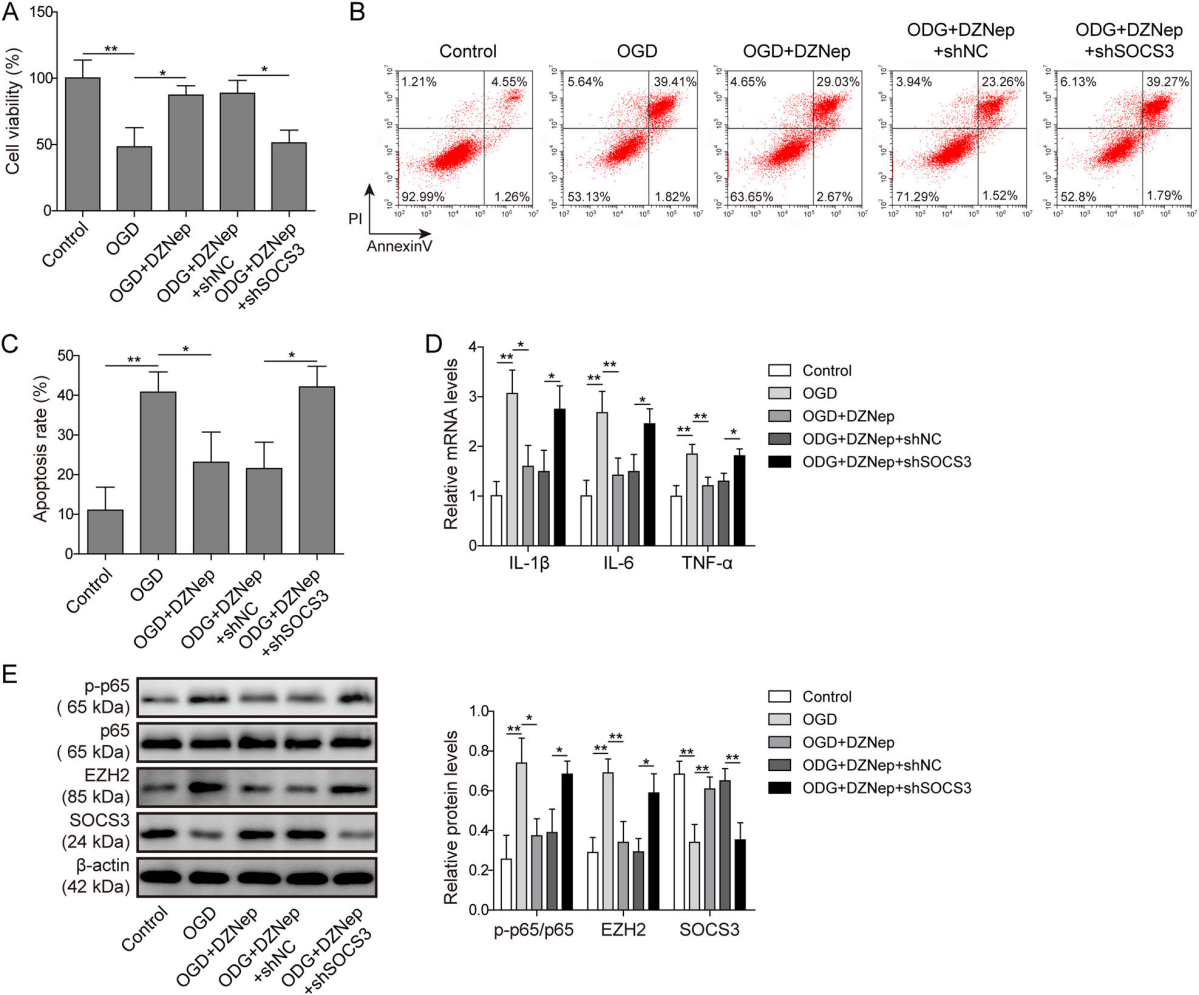
EZH2 promoted OGD-induced apoptosis and inflammation in cardiomyocytes by repressing Socs3. **a** Cardiomyocytes were assigned to five groups, i.e., no treatment (control), deprivation of oxygen and glucose (OGD), OGD treated with EZH2 inhibitor (DZNep), pre-transfected with scramble shRNA plus OGD and EZH2 inhibitor treatment (OGD + DZNep + shNC), and pre-transfected with SOCS3-specific shRNA plus OGD and EZH2 inhibitor treatment (OGD + DZNep + shSOCS3). The cell viability of each group was detected by MTT assay. **b** Apoptosis was detected by flow cytometry after annexin V/PI staining. **c** Statistical analysis of Fig. 6b. **d** The mRNA levels of IL-1β, IL-6 and TNF-α were assessed by qPCR. **e** The protein levels of p-p65, p65, H3K27me3, EZH2 and SOCS3 were determined by western blotting. The statistical analysis is presented. Error bars represent the mean ± SD. *P < 0.05 and **P < 0.01.

Given that EZH2 was shown to be a target of miR-25-3p, we explored the roles of EZH2 in OGD-induced cardiomyocyte injury. SOCS3 is one of the target genes of EZH2 and is frequently involved in cell survival and inflammatory pathways. Therefore, whether SOCS3 played a role underlying EZH2-mediated OGD events was also investigated. DZNep was used to inhibit EZH2 activity. To knock down SOCS3, shRNA selectively targeting SOCS3 (shSOCS3) or its scramble control (shNC) was transfected into cardiomyocytes prior to DZNep administration. MTT assays showed that EZH2 inhibition by DZNep partially rescued OGD-induced cell death (OGD vs OGD + DZNep, Fig. 6a). Transfection with shNC did not affect the cardioprotection-like effects of DZNep (OGD + DZNep vs OGD + DZNep + shNC, Fig. 6a). Compared with the OGD + DZNep + shNC group, knockdown of SOCS3 abolished DZNep-mediated cardioprotection after OGD (OGD + DZNep + shNC vs OGD + DZNep + shSOCS3, Fig. 6a). This result implied that SOCS3 was at least partly required for the protective effect of EZH2 inhibition. Similar findings were obtained in the apoptosis assay (Fig. 6b, c). Compared with OGD alone, DZNep administration decreased the apoptotic rates after OGD. Knockdown of SOCS3 compromised the anti-apoptotic actions of EZH2 inhibition. Moreover, EZH2 inhibition by DZNep reversed OGD-induced IL-1β, IL-6 and TNF-α upregulation. The anti-inflammatory actions were abolished by SOCS3 knockdown (Fig. 6d). Western blotting assays confirmed that DZNep efficiently reduced the upregulation of EZH2 and phosphorylation of p65 after OGD (*p* < 0.05, OGD vs OGD + DZNep, Fig. 6e), while knockdown of SOCS3 reversed these effects (*p* < 0.05, OGD + DZNep + shNC vs OGD + DZNep + shSOCS3), indicative of the presence of feedback signalling. Our data suggested that inhibition of EZH2 alleviates OGD-induced apoptosis and inflammation, while knockdown of SOCS3 reversed this protective effect of the EZH2 inhibitor.

### MSC-derived exosomes elicited cardioprotection via miR-25 in a mouse model of I/R injury

**Fig. 7:**
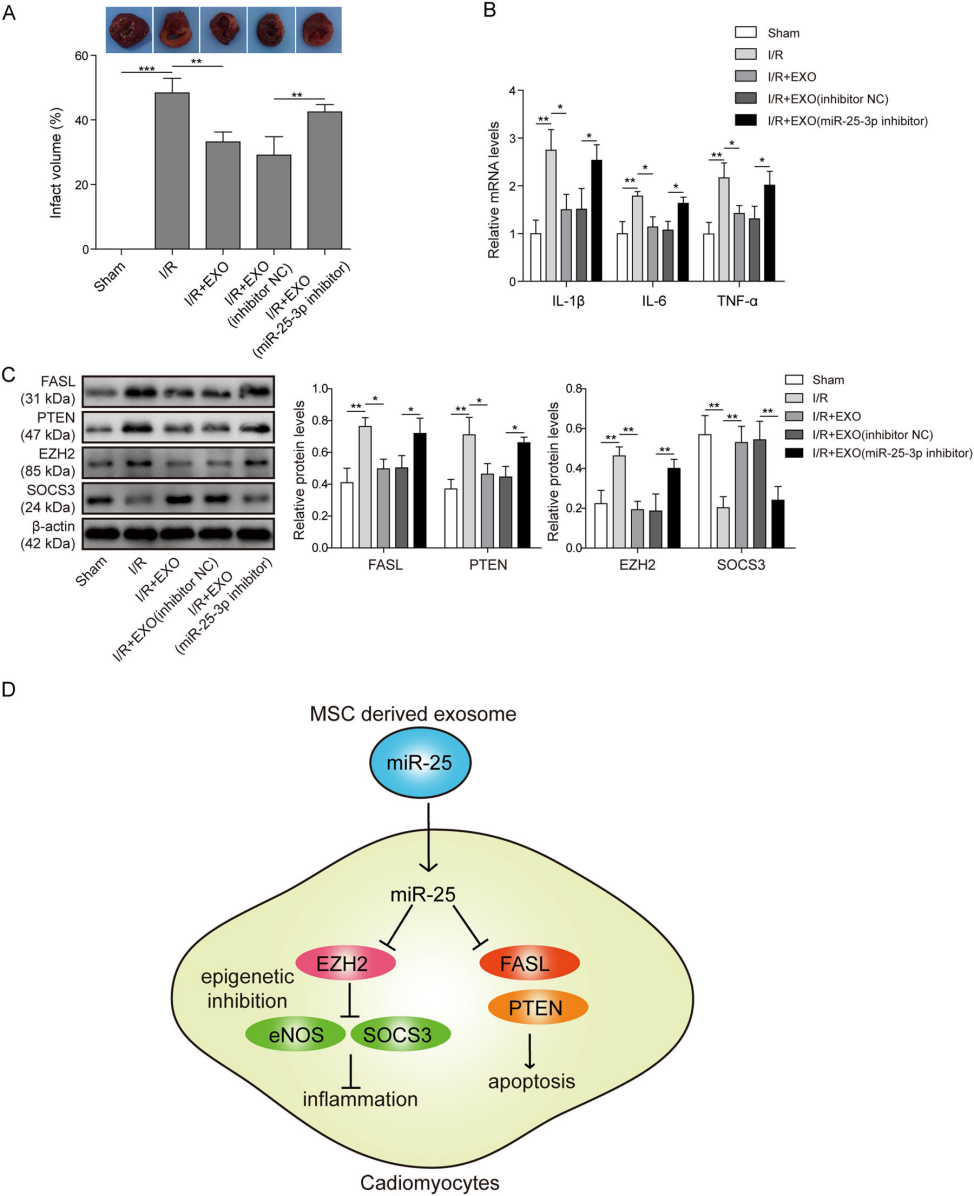
MSC-derived exosomes exhibited cardioprotection via miR-25 in a mouse model of I/R injury. **a** Mice were randomly assigned to five groups, i.e., no treatment (sham), ischaemia injury alone (I/R), I/R treated with MSC-derived exosomes (I/R + EXO), I/R injected with exosomes from scramble oligo-treated MSCs (I/R + EXO/inhibitor NC) and I/R injected with exosomes from miR-25 inhibitor-treated MSCs (I/R + EXO/miR-25 inhibitor). Representative images of tissues stained with TTC. The infarct areas from each group were compared. **b** The mRNA levels of IL-1β, IL-6 and TNF-α were assessed by qPCR. **c** The protein levels of FASL, PTEN, H3K27me3, EZH2 and SOCS3 were determined by western blotting. **d** A schematic representation of the current study. The statistical analysis is presented. Error bars represent the mean ± SD. *P < 0.05 and **P < 0.01.

The therapeutic effects of MSC-derived exosomes were explored in a mouse model of I/R injury. TTC staining demonstrated that I/R surgery induced substantial infarction in heart tissues (sham surgery vs I/R). Pre-treatment with EXO or EXO/inhibitor NC significantly reduced the infarct area compared with I/R alone (Fig. 7a). However, the therapeutic effect of exosomes was abolished when miR-25-3p was depleted in exosomes (I/R + EXO/miR-25 inhibitor vs I/R + EXO/inhibitor NC Fig. 7a). The expression of cytokines in heart tissues was also assessed. Consistent with the in vitro experiments, EXO or EXO/inhibitor NC efficiently reduced the upregulation of IL-1β, IL-6 and TNF-α after I/R (Fig. 7b). The anti-inflammatory actions of exosomes were decreased by miR-25 inhibition in exosomes (Fig. 7b). Finally, apoptosis- and inflammation-related proteins were detected in heart tissues from each group (Fig. 7c). I/R caused the accumulation of FASL, PTEN, EZH2 and H3K27me3 in injured tissues, whereas the SOCS3 level was repressed. Pre-administration of EXO or EXO/inhibitor, but not EXO/miR-25-3p inhibitor, reversed the dysregulation of these proteins, which is consistent with the findings in the in vitro assay.

## Discussion

The present study demonstrated that MSC-derived exosomes accounted for a vital part of the cardioprotective effects of MSCs by transferring miR-25-3p (Fig. 7d). By targeting FASL and PTEN, miR-25-3p reduced OGD-induced cardiomyocyte apoptosis. Moreover, miR-25-3p reversed ischaemic injury-induced EZH2 upregulation and consequently disinhibited the expression of SOCS3, leading to cardiomyocyte survival and inflammation suppression in vitro and in vivo (Fig. 7d).

Previous studies reported that the expression levels of miR-25 were associated with different subtypes of cardiovascular diseases^31^. An in vitro functional study showed that miR-25 overexpression was sufficient to protect against oxidative stress-induced cardiomyocyte death by inhibiting mitochondrial calcium upregulation^32^. In a murine model of sepsis, which induced substantial injuries to cardiac tissues, miR-25 overexpression targeted PTEN mRNA and promoted cardioprotection by counteracting apoptosis and inflammation^33^. Similar findings were obtained in the present study. MiR-25-3p overexpression by microRNA mimic transfection or exosome treatment not only facilitated cardiomyocyte survival but also suppressed detrimental inflammatory responses. In addition to PTEN inhibition, FASL suppression might also contribute to the cardioprotective effects of miR-25-3p. As these are tumour suppressor genes, it is not surprising that direct inhibition of the pro-apoptotic proteins PTEN and FASL by miR-25-3p might reduce OGD-induced cardiomyocyte apoptosis.

In addition to its anti-apoptotic action, miR-25-3p upregulation through exosome incubation led to attenuated inflammation in the in vitro and in vivo models of MI. In the present study, we provided compelling evidence that the MI therapeutic actions of miR-25-3p were achieved by directly targeting EZH2, which was a novel finding in the current study. At least two mechanisms underlying miR-25-p/EZH2 axis-mediated effects were identified. As eNOS^34–37^ confers significant cardioprotection, the finding that inhibition of EZH2 by miR-25-3p reduced methylation at H3K27 and restored the expression of eNOS represents the first molecular mechanism. Our study demonstrated a coherent mechanism by which EZH2 overactivation deteriorates ischaemia-induced tissue injury by repressing eNOS and BDNF expression in a model of limb ischaemia^27^. Numerous studies have investigated the protective roles of eNOS in different models of ischaemia. The current study focused on SOCS3 instead.

The disinhibition of SOCS3 expression could be the second mechanism. Recently, it was revealed that EZH2, through H3K27 methylation, suppressed the expression of SERPINB1, which in turn promoted inflammation-mediated prostate cancer progression^38^. This study highlighted a pro-inflammatory role of EZH2. In the current study, we reported that SOCS3 could be a target of EZH2 for inflammatory modulation. After miR-25-3p administration, EZH2 and H3K27me3 occupation at the regulatory regions of the SOCS3 promoter was significantly reduced, indicative of SOCS3 restoration. SOCS3 in turn suppressed the expression of pro-inflammatory cytokines, which may aggravate cardiac injury. These findings were consistent with previous reports that SOCS3 plays a critical role in the anti-inflammatory pathway in different scenarios of ischaemia^39,40^. Our study therefore indicated that the miR-25/EZH2/SOCS3 axis was a critical inflammation modulatory network of MI and that EZH2 was the upstream regulator of SOCS3 acting as a potential target for MI therapy. It should be noted that several papers have reported a deleterious role of SOCS3 in a conditional knockout mouse model^41,42^. In these studies, the molecular function of SOCS3 has been closely linked to apoptosis in two mouse models of MI. Our study, however, revealed that SOCS3 played a pivotal role in exogenous miR-25 transfer (via MSC-derived exosomes) or EZH2 inhibitor-mediated suppression of inflammation, which is a crucial component of I/R aetiology. The anti-inflammatory roles of SOCS3 are consistent with most previous studies^43,44^. With respect to apoptosis, it is noteworthy that both miR-25 and EZH2 inhibitor reduced a series of pro-apoptotic genes, including Fas and PTEN, by reshaping the OGD-induced epigenome. Such alterations may counteract pro-apoptotic signalling in our model. Moreover, upregulation of SOCS3 has been considered to benefit animals or cells by suppressing inflammation and hence conferring anti-apoptotic effects in other ischaemia models^45,46^. We concluded that SOCS3 may play different roles under specific circumstances (i.e., initiation of I/R injury vs. therapy) or in specific components of aetiology (i.e., apoptosis vs. inflammation). In addition, contradictory roles for EZH2 in inflammation have also been reported. For instance, degradation of EZH2 by E3 ligase was demonstrated to facilitate recruitment of pro-inflammatory macrophages into colon tissues^47^. These studies implied that EZH2 exhibited distinct functionalities in different contexts of pathological alterations. More detailed studies are required prior to attempts to target EZH2-related signalling networks for the treatment of different diseases.

In the current study, we first demonstrated that MSCs were sufficient to reduce OGD-induced cell apoptosis and pro-inflammatory responses. Next, we found that exosomes secreted by MSCs accounted for a vital part of the cardioprotective effects of MSCs in vitro and in vivo. Moreover, we revealed that one of the exosomal cargoes, miR-25-3p, drove these effects of MSCs. Depletion of miR-25-3p in exosomes abolished the biological actions of these exosomes in vitro and in vivo. Hence, we propose that delivery of miR-25-3p to cardiomyocytes via exosomes represents another mechanism underlying MSC-mediated cardioprotection. Furthermore, our study suggested that the preparation of miR-25-3p-containing exosomes could be a novel approach for the treatment of MI.

## Supplementary information

supplemental figure legends

figure S1

figure S2
