# Correction: Exosomal miR-25-3p from mesenchymal stem cells alleviates myocardial infarction by targeting proapoptotic proteins and EZH2

**DOI:** 10.1038/s41419-020-02996-8

**Published:** 2020-09-23

**Authors:** Yi Peng, Ji-Ling Zhao, Zhi-Yong Peng, Wei-Fang Xu, Guo-Long Yu

**Affiliations:** Department of Cardiology, Xiangya Hospital, Central South University, Changsha, 410008 Hunan Province P.R. China

**Keywords:** Cell death, Cell death, Cardiovascular diseases, Cardiovascular diseases

Correction to: *Cell Death & Disease*

10.1038/s41419-020-2545-6, published online 5 May 2020

Since online publication of this article, the authors noticed that an incorrect image was used during the compilation of Fig. 2a, which was caused during manuscript preparation. The correct Fig. 2a is shown on the next page.

**Figure:**
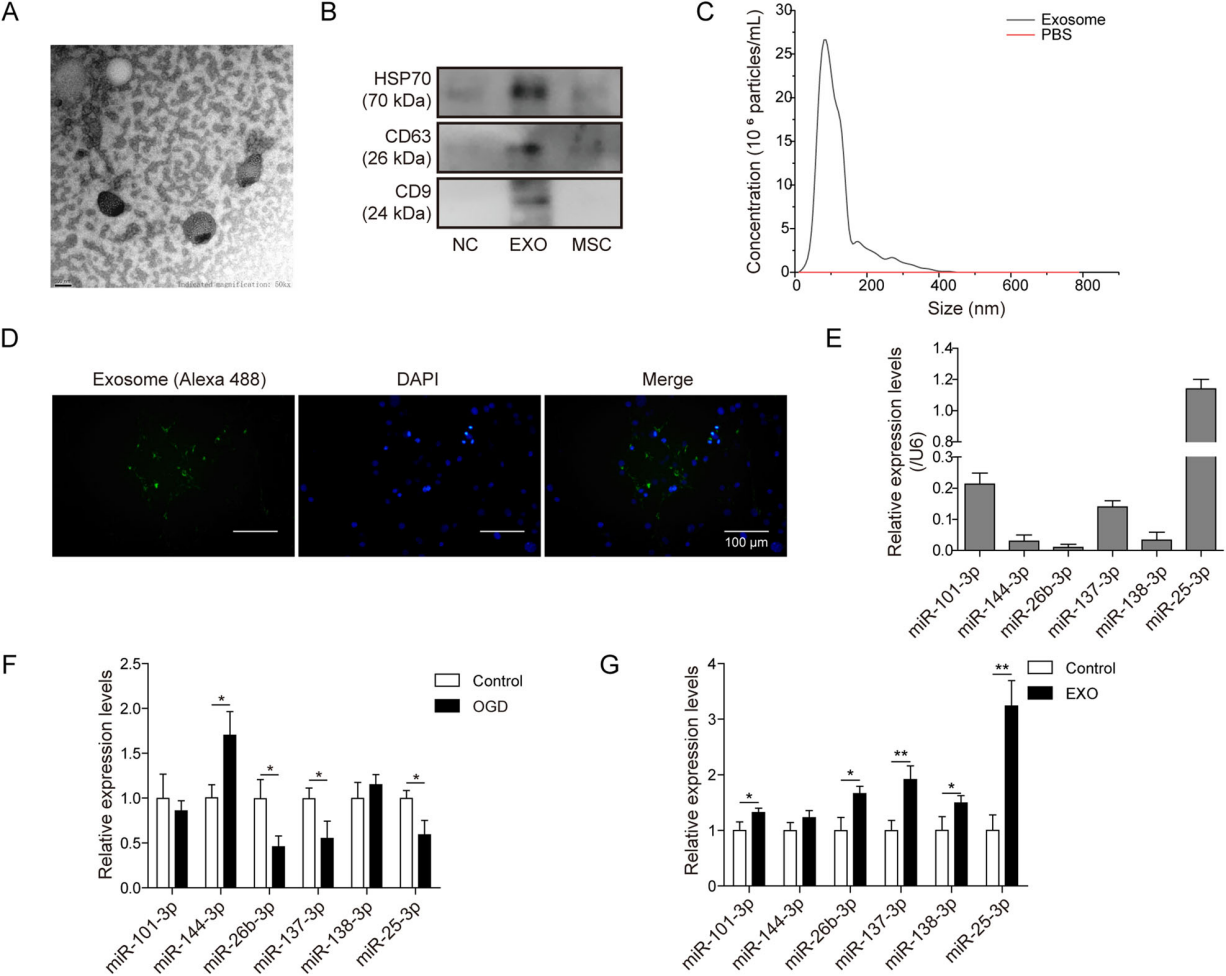
Fig. 2.

In addition, in the last sentence of the fifth paragraph of the “Materials and methods” section, the sentence “The cells co-labelling with Maleimide (green) and cell tracker (red) under the confocal microscope were considered as positive cells containing exosomes” should read “The cells labelling with Alexa 488 (green) under the microscope were considered as positive cells containing exosomes”.

The authors apologise for these errors, and confirm that this does not affect the conclusions of the study.

