# Correction: Exosomal miR-25-3p from mesenchymal stem cells alleviates myocardial infarction by targeting pro-apoptotic proteins and EZH2

**DOI:** 10.1038/s41419-020-03025-4

**Published:** 2020-10-12

**Authors:** Yi Peng, Ji-Ling Zhao, Zhi-Yong Peng, Wei-Fang Xu, Guo-Long Yu

**Affiliations:** Department of Cardiology, Xiangya Hospital, Central South University, Changsha, 410008 Hunan Province P.R. China

**Keywords:** Cell death, Cardiovascular diseases

Correction to: *Cell Death & Disease*

10.1038/s41419-020-2545-6 published online 5 May 2020

Since online publication of this article, the authors noticed that an incorrect image was used in Fig. 2a. The corrected Fig. 2 has been provided below.

**Fig. 2:**
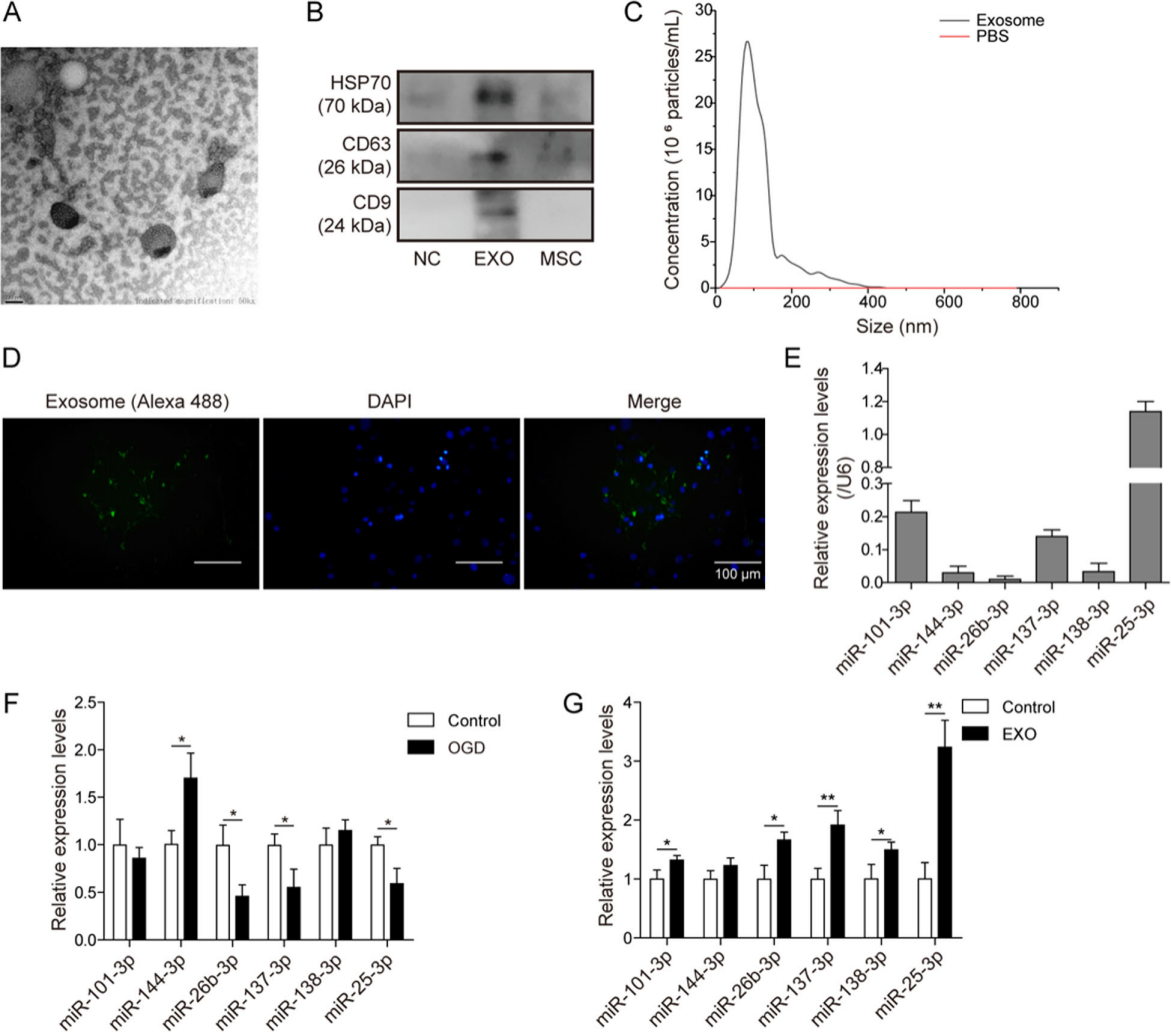
▮.

In addition, the last sentence of the “Material and methods” section describing the *Detection of exosomes uptake by cardiomyocytes* contained incorrect information on labelling. The full and corrected paragraph is provided below.

Isolated exosomes were incubated with 3.3 μL of Alexa FlourTM 488 C5 Maleimide (200 μg/mL, A10254, Thermo Scientific, San Jose, CA, USA) for 1 h at room temperature. The labelling was disturbed by passing through the exosome spin column (MW3000, 4484449, Thermo Scientific, San Jose, CA, USA), according to manufacturer’s instruction. The labelled exosomes were washed out and resuspended with 1 mL of serum free OptiMEM (31985088, Thermo Scientific, San Jose, CA, USA). For each well in a 4-well plate, 250 μL labelled exosomes were incubated with primary cardiomyocytes in the standard cell culture condition for 4 h at 37 °C. Cardiomyocytes were then counterstained with CellTracker Deep Red dye and mounted with ProLong Gold antifade mountants without DAPI (#P36934, Thermo Scientific, San Jose, CA, USA). The cells labelling with Alexa 488 (green) under the microscope were considered as positive cells containing exosomes.

The authors apologise for these errors.

