# Supplementary material for: Exosomal miR-25-3p from mesenchymal stem cells alleviates myocardial infarction by targeting pro-apoptotic proteins and EZH2
Source: Cell Death Dis. 2020 May 5;11(5):317. doi: 10.1038/s41419-020-2545-6 (PMC7200668; doi:10.1038/s41419-020-2545-6)
Supplement: Supplementary file 1 — supplemental figure legends [file 41419_2020_2545_MOESM1_ESM.docx]

**Figure S1. Effects of miR-25 inhibitor on the expression of pro-apoptotic genes.** **A.** Cells were transfected with miR-25 inhibitor or scramble control. RT-PCR demonstrated that compared with the scramble control, the miR-25 inhibitor significantly reduced the RNA level of miR-25-3p in cardiomyocytes. **B.** miR-25 downregulation was accompanied by upregulation of FASL, PTEN and EZH2 mRNA levels. Error bars represent the mean ± SD. * P<0.05 and ** P<0.01.

**Figure S2. Effects of MSC-derived exosomes on 3’UTR luciferase activity.** The addition of MSC-derived exosomes was sufficient to reduce the luminescence of luciferase fused with the 3’UTR of FASL, PTEN or EZH2. Error bars represent the mean ± SD. * P<0.05 and ** P<0.01.
