# Supplementary figures and images for: Exosomal miR-25-3p from mesenchymal stem cells alleviates myocardial infarction by targeting pro-apoptotic proteins and EZH2
Source: Cell Death Dis. 2020 May 5;11(5):317. doi: 10.1038/s41419-020-2545-6 (PMC7200668; doi:10.1038/s41419-020-2545-6)

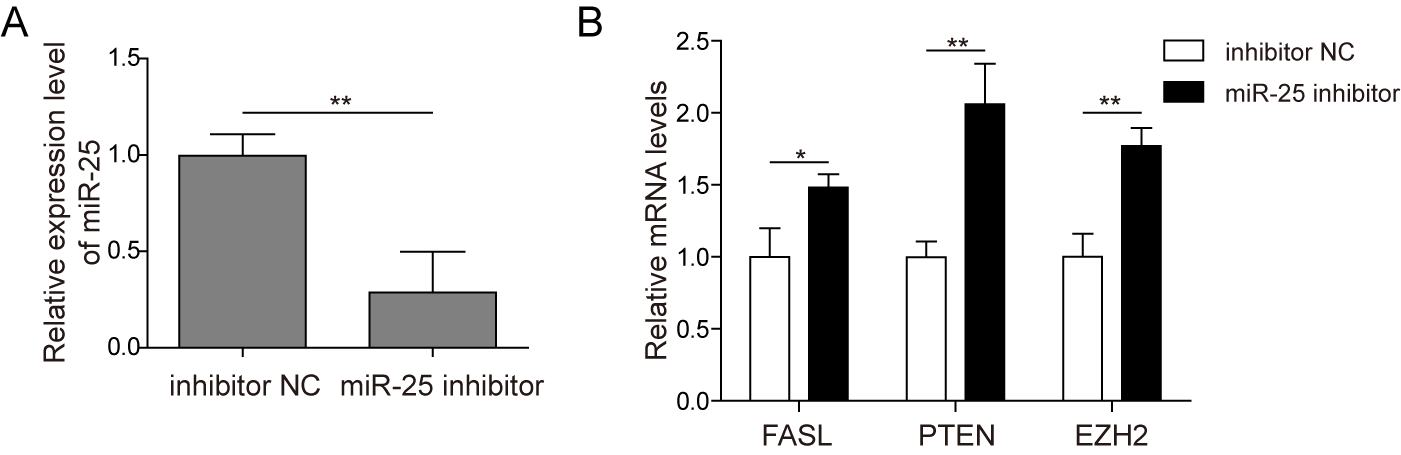

Supplement: Supplementary file 2 — figure S1 [file 41419_2020_2545_MOESM2_ESM.tif]

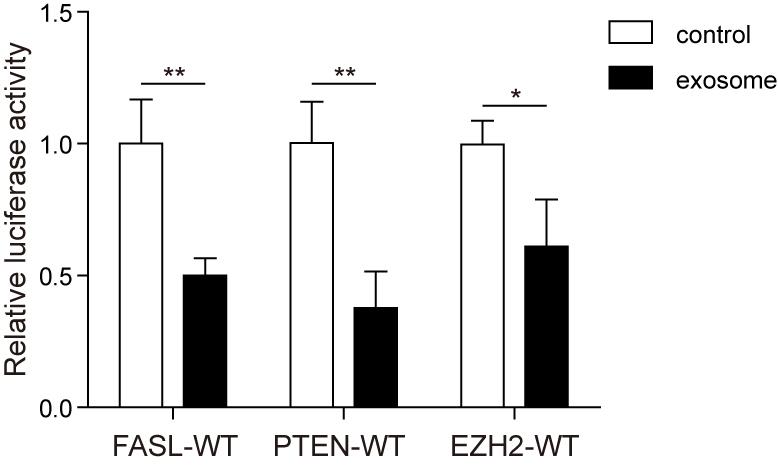

Supplement: Supplementary file 3 — figure S2 [file 41419_2020_2545_MOESM3_ESM.tif]
